# Electronic health record implementation: how to identify and analyze the possible negative impacts

**DOI:** 10.31744/einstein_journal/2024AO0916

**Published:** 2024-11-12

**Authors:** Paula Fuscaldo Calderon, Silvia Sato, Nelson Wolosker

**Affiliations:** 1 Hospital Israelita Albert Einstein São Paulo SP Brazil Hospital Israelita Albert Einstein, São Paulo, SP, Brazil.; 2 Accenture São Paulo SP Brazil Accenture, São Paulo, SP, Brazil.

**Keywords:** Electronic health records, Medical records, Medical informatics, Change management, Health evaluation, Information technology, Health personnel, Models, organizational

## Abstract

**Objective:**

Identify potential negative impacts arising from implementing an electronic medical record system, classify them according to the level of criticality, and analyze method’s effectiveness after implementation.

**Methods:**

The research involved identifying the negative impacts, classifying them according to the criteria for criticality, stratifying them as high, medium, or low severity, and finally, analyzing the effectiveness of the identification and classification methods.

**Results:**

Findings confirmed that 89.20% of identified impacts occurred as predicted, and 88.94% of impacts had a level of criticality compatible with the severity of the problem.

**Conclusion:**

Predicting and classifying negative impacts are important stages in implementing electronic health records in hospitals. The method for identification and classification of impacts were, in most cases, considered effective.

## In Brief

Hospitals are increasingly acquiring information systems that require a change management method. Calderon et al. identified the potential negative impacts resulting from the implementation of an electronic medical record system and classified them according to criticality to assist in project management and ensure successful implementation.

## Highlights

■ The electronic health record is the first step in digital transformation.

■ Change Management is essential to support problems identification.

■ Identifying and stratifying negative impacts within the staff’s daily routine is crucial.

■ A new method was created, and the method’s effectiveness was evaluated.

## Graphical abstract

**Figure:**
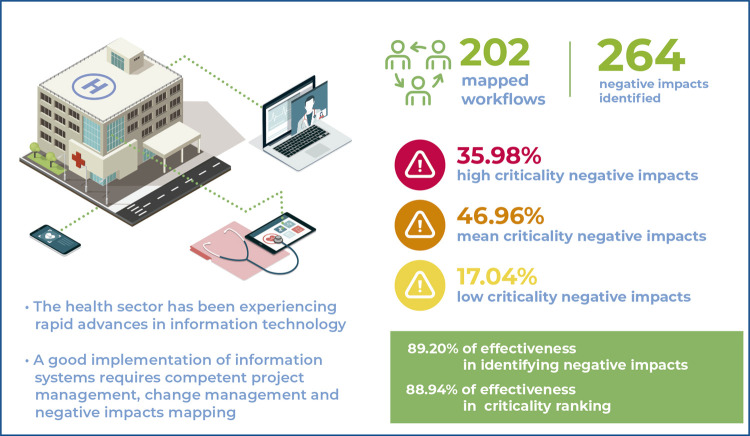

## INTRODUCTION

Digital technologies are being widely adopted in the healthcare industry, leading to a widespread transformation of the sector.^(1)^ The use of information technology in hospitals has proven to be a major tool for improving the quality and safety of services provided and reducing costs.^(2-5)^ In health services, implementing an electronic health record (EHR) is the first step in the improvement process to promote operational efficiency through technology. There is a significant concern with EHR implementation due to the high failure rates observed in the literature, which can reach rates higher than 50%.^(6)^ For good EHR acceptance by users, the new tool must be adapted to the processes and workflows of the professionals who work in the institution,^(5,7)^ and the organization’s leadership support is essential.^(8-10)^

Users have a natural tendency to resist^(10)^ due to concerns about changes in clinical practice and increased workload.^(11-15)^ These concerns are directly related to the daily task execution processes, and the most critical moment is immediately after implementation because it requires a more significant cognitive effort from users to adapt and perform the same activities they performed before but now using a new tool. If the system is not adapted and causes disruption in the linear workflow, it will automatically lead to user dissatisfaction and may cause platform rejection, compromising system adoption and continued use.^(5,14,16)^

So, despite the EHR’s positive impacts, it takes good governance, which requires management of change and a suitable project management method to manage the negative impacts. In the information technology (IT) industry, the project management methodology PMBOK (Project Management Body of Knowledge) published by the American “Project Management Institute” is widely used for system implementation^(17-22)^ but is still underreported in the medical literature. In the PMBOK guide, a specific step recommends the risk treatment and project impacts.^(23)^ New projects are idealized and implemented because they improve and benefit the institution, patients, and health professionals. They also generate negative impacts, especially in the staff’s daily routine, which should be identified and addressed during the pre-implementation phase.^(6,8,11)^ To the best of our knowledge, no studies have demonstrated the usefulness of the methods to identify and analyze the potential negative impacts before the implementation of EHR in hospitals.

## OBJECTIVE

The objective of this study was to identify the possible negative impacts, for the users in the new electronic health record incorporation process in a quaternary hospital, as well as to stratify these problems according to severity, thus contributing to decision-making regarding preventive actions. After the software implementation, the study also aimed to evaluate the identification method’s effectiveness and classification of these impacts.

## METHODS

This is a retrospective and descriptive study. The Millenium software, from the company CERNER^®^, version 2015.01.09, Kansas, Missouri, USA, was chosen for the EHR implementation project in all care areas of a hospital complex with 750 beds, one outpatient unit containing a day hospital associated with an outpatient clinic, and emergency care (urgency and emergency) and two outpatient emergency care units (urgency and emergency), with 15,000 employees, 2,000 contracted physicians, and 8,000 open clinical staff physicians. The project started in January 2014, and implementation (Go Live) first occurred in October 2016 in the two emergency care units, next, in November 2016, in the outpatient unit with day hospital, outpatient clinic, and emergency care, and in January 2017 in the hospital complex.

Thirty-eight professionals considered to be referenced in their work fields and the hospital’s different stakeholder representatives, who had institutional hospital practices’ in-depth knowledge, were selected and hired to work exclusively on the EHR implementation project.

The group of contracted professionals was sized as follows: 10 physicians (1 Nephrologist, 2 Geriatricians, 1 Cardiologist and Intensivist, 1 Anesthesiologist, 1 Oncologist, 2 Pediatricians, 1 Neonatologist, 1 Hematologist and Emergency Room); 9 nurses representing the hospital main sectors (1 Adult ICU, 1 Child ICU, 1 Neonatal ICU, 1 Maternity, 1 Emergency Care Units, 1 Oncology, 1 Surgical Center, 1 Inpatient Units and 1 Outpatient Units); 2 Biomedicals (representing the Image exams and Laboratory sectors); 2 Physiotherapists, 1 Speech Therapist, 1 Nutritionist (representing the entire hospital’s multi-professional team); 4 pharmacists, 3 registration area professionals; 4 financial professionals, 2 commercial professionals. For analysis purposes, these professionals were grouped into six major groups: physicians, nurses, a multi-professional team (nutritionists, physical therapists, speech therapists), pharmacists, biomedicals and an administrative team (financial, commercial, and registration).

The key users mapped the main work processes, also called workflows, of each sector of the hospital, considering the most frequent processes that covered most of that department’s daily activities, to compare the workflows of the areas before the EHR implementation with the workflows after the EHR implementation and identify the changes. Any change in workflow due to the EHR implementation would result in an impact on the professionals working in that workflow. The impact could be positive or negative. For this study, only negative impacts were considered.

From August 2014 to September 2016, key users identified and stratified impacts according to their criticality.

First, the key users reviewed all workflows in weekly meetings and discussed all steps of each workflow, aiming to identify changes. An impact was identified if there was a change in any of the steps because of the software implementation. There was no rule for detecting impacts per workflow, and anything from no impact to an unlimited number could have been detected.

Then, each impact was classified according to the problem severity (criticality). The criticality was classified based on 5 criteria, shown in table 1.

**Table 1:** Criticality criteria

Criteria	Description
Cultural change	Activity that, due to the system implementation, forced the user to change his way of thinking and acting.
Quality and safety	Complex activity within system unfavorable to usability that could lead to user errors, which could compromise the safety and patient care quality.
Institution image	Activity that misleads the user or slows down the process by increasing patient waiting time may result in interest loss or patient confidence loss.
Work time increase	Activity that would increase the total time spent to perform a given task, but with a tendency to normalize as the system is learned and used.
Tool adaptation	New activity due to the system characteristics and the user would be able to adapt quickly with training.

The impacts were stratified into 3 levels of criticality: high, medium, or low. High criticality impacts were considered those that could compromise the quality and safety of patient care and/or compromise the institution’s image and/or incur a cultural change. Medium criticality impacts were considered when, excluding high criticality criteria, those requiring more time to perform the activity. Low criticality impacts were considered when, excluding high and medium criticality criteria, those in which the change would be quickly accepted through training users to utilize the tool, as described in table 2.

**Table 2:** Criticality impacts ranking

Criteria	Criticality high	Criticality mean	Criticality low
Cultural change	Yes	No	No
Quality and safety	Yes	No	No
Institution image	Yes	No	No
Work time increase	-	Yes	No
Tool adaptation	-	-	Yes

Finally, 6 months after system implementation in the hospital complex, for the expected impacts that were not solved in project time and therefore occurred during Go Live, the teams that participated in the project analyzed whether: 1) The identified impact was confirmed or not; 2) The criticality assigned was compatible with the problem severity or not.

In statistical analysis absolute numbers and frequency were used to analyze the data. The frequency data were presented as a percentage. Thus, we obtained the dimension of the total impacts identified and their criticality, and it was also possible to analyze the true effectiveness of the methods used for identification and severity classification.

The exclusion criteria for analysis of this research were: 1) lack of sufficient information for the analyses, 2) positive impacts, and 3) impacts identified for functionalities or solutions offered by the software that were not implemented.

### Ethical considerations

The data analyzed in this study does not involve experiments on humans and/or human tissues, nor does it disclose the personal information of individuals. Therefore, the Ethics Committee of *Hospital Israelita Albert Einstein* waived ethical approval and informed consent to publication.

## RESULTS

Two hundred and two workflows were mapped, within which 264 negative impacts were identified or predicted as shown in Table 1S, Supplementary Material. The distribution of the 264 negative impacts according to the professional groups that would be affected by the changes is presented in table 3. Most identified impacts occurred in the physician’s workflow, followed by nurses. The criticality distribution of the 264 impacts occurred according to table 4. Most of the impacts were considered medium criticality, but more than a third were highly critical.

**Table 3:** Descriptive analysis of the total negative impacts identified according to professional category

Category	Impacts number n (%)
Physicians	134 (50.75)
Nurses	71 (26.89)
Multi-professional team	19 (07.19)
Biomedicals	16 (06.06)
Pharmacists	08 (03.03)
Administrative team	16 (06.06)
Total	264 (100)

**Table 4:** Descriptive analysis of the total negative impacts according to problem severity or criticality

Criticality	Impacts number n (%)
High	95 (35.98)
Mean	124 (46.96)
Low	45 (17.06)
Total	264 (100)

Out of the total 264 impacts identified, 51 were resolved during the project and were not part of the following analyses. The 213 negative impacts distribution, which would happen at Go Live, according to the groups of professionals that would be affected by the changes, is presented in table 5. The proportion remained the same and most of the identified impacts occurred in the physician’s workflow, followed by nurses. The criticality distribution of the 213 negative impacts, which would happen at Go Live, occurred according to table 6. Similarly, the proportion remained the same, and most impacts were considered of medium criticality, and more than a third of the impacts were highly critical.

**Table 5:** Descriptive analysis of the negative impacts, which would happen at Go Live, according to professional category

Professional category	Impacts number n (%)
Physicians	97 (45.53)
Nurses	64 (30.04)
Multi-professional team	19 (08.92)
Biomedicals	13 (06.10)
Pharmacists	08 (03.75)
Administrative team	12 (05.63)
Total	213 (100)

**Table 6:** Descriptive analysis of the negative impacts, which would happen at Go Live, according to problem severity or criticality

Criticality	Impacts number n (%)
High	73 (34.27)
Mean	100 (46.94)
Low	40 (18.77)
Total	213 (100)

**Figure 1:**
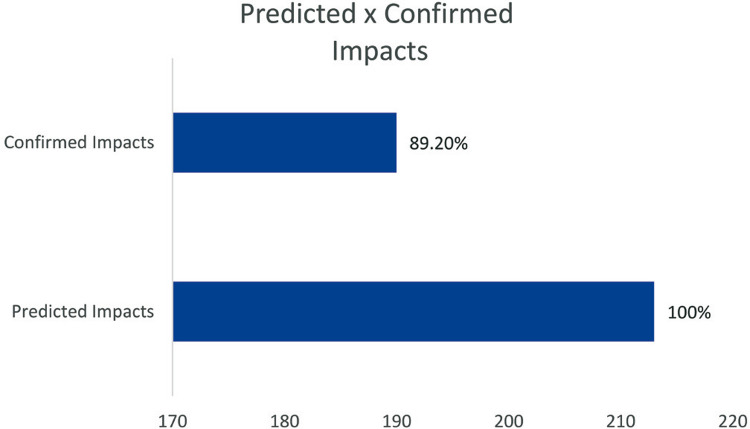
Comparative analysis between predicted versus confirmed impacts

Six months after Go Live at the hospital complex, key users were interviewed about the 213 unresolved impacts at the design time, which were left to be mitigated at Go Live. Then, it was analyzed whether or not the predicted impact was confirmed and shown in figure 1 and in Table 2S, Supplementary Material. Most of the impacts identified at design time were real (89.20%), and the minority were not confirmed. Under the 190 impacts confirmed at Go Live, it was analyzed whether the predicted criticality was compatible with the problem severity or not (Table 7). The impact criticality was compatible with the severity of the problem in 88.94% of the cases.

**Table 7:** Confirmation of problem severity or criticality according to negative impacts

Criticality	Impacts number n (%)
Confirmed	169 (88.94)
Not confirmed	21 (11.05)
Total	190 (100)

## DISCUSSION

Information technology is revolutionizing the health sector and has great potential to improve processes, increase the quality of services, and reduce costs.^(4)^ However, to ensure all these positive impacts, these technologies have a good implementation plan and execution, which includes, within the planning, the negative impacts mapping.^(8)^ Specifically, in the case of electronic health records in hospitals, user participation is essential for the actions to be effective.

Usually, information system implementation projects involve the information technology professionals’ participation and also key users from different corporation business areas only as a support and guidance team. Still, the latter are often not fully dedicated, hindering their effective contribution.^(1)^

In this project, a planning process was carried out in which representatives of the hospital’s different stakeholders, with deep knowledge of their activity areas, were selected key users. These professionals were relocated from their hospital sectors and were hired to work full-time exclusively for this electronic medical record software implementation.

Key users were not only involved as support in the system configurations performed by the IT team but were actively engaged in adapting the software to the workflows. To this end, the teams mapped the main institution workflows. Obviously, a highly complex hospital has many work processes in each business area, where the most frequent processes affect that particular sector’s daily routine, and the less frequent or exceptional processes that do not interfere with the daily routine. So, only the most frequent workflows were mapped, totaling 202 to be analyzed and adapted.

During mapping and adjusting the flows, the key users were oriented and trained to identify and notify the change points that could have a problem or negative impact on the end-users. Predicting possible issues that could happen to the tool’s end users was not easy. After all, the 100% routine of the institution’s employees would change. However, this became possible due to the precise method for identifying the impacts. This way, 264 change points were identified and recognized as negative impacts of the software implementation.

Due to the project’s magnitude and the institution’s size, both in terms of the physical structure and the number of employees involved, it was imagined at the project´s beginning that the number of impacts identified would be much higher. Still, the analysis depended on the individual perception of each key user. Even so, the most impactful problems that could compromise the implementation were duly identified.

Faced with the number of problems identified and the possible preventive actions that could be performed to try to solve them, the question that naturally arose was, “Where to start?” Due to the infeasibility of treating all the identified problems simultaneously, it was necessary to establish some sort of criteria.

The PMBOK guide recommends mapping risks and impacts, as well as stratifying them into high, medium, or low to direct resolution efforts. Stratification is usually done through event occurrence probability analysis.^(23)^ In this work, impacts were stratified into high, medium, or low; however, since this is a healthcare institution, what is considered critical is not necessarily related to the frequency with which an episode occurs. For example, in the case of a critical test result or a serology test, if the software functionality is difficult to the point that the laboratory employee makes a mistake in recording the result, the consequences for the patient and the institution can be catastrophic, regardless of the likelihood that this test is performed on a daily or monthly basis. Therefore, a particular activity may happen only a few times, but the risk is high for the patient and the institution if there is an error.

Thus, in this work, the traditional risk and impact matrix methods would not apply because they use the frequency of the events to establish criticality. So, new criteria were created considering what would be critical for the patient (quality and safety), the institution (image), and the staff (cultural change), and these were the considered high severity criteria. It is known that in system implementation, there is a learning curve for users; thus, during a period, users take longer than usual to perform the same tasks, and this condition was considered a medium severity criterion. Finally, in the case of a change requiring only adaptation to the tool from the collaborators, as long as high and medium severity criteria were excluded, they were considered low severity criteria. Again, the key users played an important role in this step because they helped classify the severity of these impacts according to the pre-established criteria and thus contributed to the project management regarding decision-making for preventive actions.

After software implementation, the study also aimed to evaluate the effectiveness of identification and classification method of impacts. The Go Live took place on a previously scheduled date, and we waited six months after implementation in the hospital complex for stability, process accommodation, and the users’ learning curve. After this period, the effectiveness of the impact identification and the criticality classification methods was analyzed. The problems considered fully resolved before the implementation were not part of this analysis. Then, the 213 remaining impacts that would occur at Go Live were revisited and analyzed one by one in a joint meeting with key users. In the opinion of the key users, the impact identification method was effective in 89.20% of the cases, and the criticality ranking was effective in 88.94%.

### Study limitations

The analysis period of the method’s effectiveness occurred six months after the system’s implementation, and adding the three years of project time, we arrived at a total period of three and a half years. Due to the long time between the project, implementation, and accommodation of the software, it is expected that some people who participated in the project will no longer be in the institution after this period, because there is a natural turnover of staff in corporations. So, when the teams that participated in the project were summoned for the analysis meetings, some employees were no longer part of the corporation, which may have interfered with the method’s effectiveness results.

During the project, the process of identifying and stratifying the impacts was done in a group, and the decision was collegiate. Thus, the post-implementation evaluation was done in the same model, where the teams participated in the meetings and jointly decided on the answers. Instead, an individual questionnaire could have been applied to analyze the results. This group analysis model may have interfered with the effectiveness method results.

## CONCLUSION

Predicting negative impacts is an important step in implementing electronic medical records in hospitals, which becomes feasible when using a methodology based on the group activity of a dedicated multi-professional team. These impacts can then be classified according to their criticality degree, in conformity with what is considered critical to the institution. Their analysis makes it possible to establish preventive action plans. Identifying and classifying the impacts was considered effective in most cases.
