# Supplementary material for: Electronic health record implementation: how to identify and analyze the possible negative impacts
Source: Einstein (Sao Paulo). 2024 Nov 12;22:eAO0916. doi: 10.31744/einstein_journal/2024AO0916 (PMC11634333; doi:10.31744/einstein_journal/2024AO0916)
Supplement: Supplementary file 1 [file 2317-6385-eins-22-eAO0916-suppl01.pdf]

SUPPLEMENTARY MATERIAL

Electronic health record implementation: how to identify and analyze the possible negative impacts

Paula Fuscaldo Calderon, Silvia Sato, Nelson Wolosker

DOI: 10.31744/einstein_journal/2024AO0916

Table 1S. Description of impact according to professional group, criticality level and status

	Professional group	Description of impact	Criticality level	Status
1	Physicians	The continuous infusions prescription process in the system is culturally completely different, because the American system does it in a very different way from the way it is done in Brazil. In the system, continuous infusions do not require setting up a frequency, and all that is necessary is informing the infusion time.	High	Go Live Mitigation
2	Physicians	Process change for the physician regarding the act of prescribing medications, because the medication administration time is automatically scheduled, so the physician needs to be instructed on how the frequencies available in the system for prescribing medication operate to avoid problems for the patients.	High	Go Live Mitigation
3	Physicians	The act of saving documents in the system requires filling in a number of fields, which burdens the physician's time.	High	Go Live Mitigation
4	Physicians	The medication prescription "at the physician's discretion" is cultural and thus the system had to be adapted to absorb this type of prescription. The physician will need to be trained because due to the adaptation the process is different from the usual for the physician, and they will have to input the information "at the physician's discretion" into two different fields.	High	Go Live Mitigation
5	Physicians	System screens with patient information can receive new information all the time, so the physician will have to get used to refreshing the screen to receive new information. New physician activity.	High	Go Live Mitigation
6	Physicians	The registration of diagnostic hypotheses or confirmed diagnoses is complex, requires many clicks, burdens the physician's time, and requires training.	High	Go Live Mitigation
7	Physicians	The physician usually notes many of the patient's health conditions on the patient record that are not necessarily in the ICD table, so on the electronical health record it will be difficult to record this information in the field defined for diagnoses.	High	Go Live Mitigation
8	Physicians	The structure of prescription viewing is culturally completely different from what is done at present, and physicians will need to get used to this new way of viewing prescriptions.	High	Go Live Mitigation
9	Physicians	In the electronic health record, the record of difficult airway needs to be done using the ICD table, which is a culture change for the physician.	High	Go Live Mitigation
10	Physicians	New surgeon physician activity to activate prescription package for medications administered in the surgical center.	High	Go Live Mitigation
11	Physicians	Viewing the dialysis patients serologies is not easy; the physician will have to search for the information, which is time-consuming. This information is primordial in the dialysis sector.	High	Go Live Mitigation
12	Physicians	One more change in the dialysis patient monthly report, where physicians will have to attach the multidisciplinary team reports to their own report to provide a copy for the patient.	High	Go Live Mitigation
13	Physicians	New process for the physician due to the way the system works. In ICU shifts where the physician is responsible for a particular patient for 12 hours, they will have to assign themselves as responsible for the patient in the system, and after the shift is over, they will have to unassign themselves.	High	Go Live Mitigation
14	Physicians	The way of recording all the patient's personal history into the system is completely different from the process physicians are already used to, and the information is fragmented, separated into structured fields that must be filled in separately with many clicks in the system.	High	Go Live Mitigation
15	Physicians	There is a feature in the system that is prescription packages, which makes the physician's day-to-day life easier. These packages can be planned, and even though planning is a benefit, these prescription packages need to be activated to become effective, which is a completely new activity due to the system change.	High	Go Live Mitigation
16	Physicians	Physician cannot forget to save the clinical document because if they forget and move on to another patient in the system or log out, all information will be lost.	High	Go Live Mitigation
17	Physicians	Cultural change in the way pediatric physicians work, as they generally prescribe dosages expressed in terms of mg/kilo/day, whereas the system only displays kilo/dose associated with the medication frequency.	High	Go Live Mitigation
18	Physicians	The way prescriptions for compounded medications with two drugs are written is different for pediatrics and for adults. However, the system only allows one presentation form, so either pediatricians or clinicians will have to adapt.	High	Go Live Mitigation
19	Physicians	Neonatologists will have to adapt to the process change in the clinical data registration, especially at the discharge time from the Neonatology ICU when it will be necessary to register two documents in the system. One will be the daily evolution and the other will be the discharge documentation. In system, these two documents are separated and need to be created and saved.	High	Go Live Mitigation

continue...

...Continuation

Table 1S. Description of impact according to professional group, criticality level and status

	Professional group	Description of impact	Criticality level	Status
20	Physicians	Change in the APGAR recording process for neonatologists who will need to adapt to the new process within the system.	High	Go Live Mitigation
21	Physicians	Gynecologist's process change in recording gynecological history, as information is separated in different structured fields.	High	Go Live Mitigation
22	Physicians	The visualization of conclusion laboratory test reports is extremely difficult, because it is necessary to change screens, burdening the physician in search time.	High	Go Live Mitigation
23	Physicians	The visualization of test results with a curve, for example a glycemic curve, is different from what physicians are culturally used to.	High	Go Live Mitigation
24	Physicians	Process change for gynecological physicians in recording the number of pregnancies of a patient, where there will be a specific structured field for inputing each pregnancy and the system automatically calculates the number.	High	Go Live Mitigation
25	Physicians	There will be a process change for physician surgeons due to a new functionality in system, which provides ready-to-use prescription packages with antibiotic prophylaxis for surgeries.	High	Go Live Mitigation
26	Physicians	The family history register in the system is through structured fields, and when there is the need to register information that is not structured, the physician will have to make a path through the system with many clicks, burdening the physician's time.	High	Go Live Mitigation
27	Physicians	Physicians will need to be trained to order the required lab tests and sign the order only at the end, so as not to generate multiple test codes that can impact the lab. Therefore, physicians will need to be trained.	High	Go Live Mitigation
28	Physicians	New process for the nephrologist physician who will have to record the service type in the system at the time of the dialysis prescription, that is, if it is an outpatient dialysis prescription or if it is an inpatient dialysis prescription.	High	Go Live Mitigation
29	Physicians	Process change for the physician who will have to validate the prescription in the system every 24 hours. The process was adapted because of the culture in Brazil.	High	Go Live Mitigation
30	Physicians	In the system, each patient has a personal chart number, but in every visit to the hospital, a number is generated for that new visit. Physicians need to be warned and trained not to put information in the wrong visit number.	High	Go Live Mitigation
31	Physicians	For the patient level of care transfer, there is a screen to be filled out for medication reconciliation. The new activity increases the physician's workload.	Medium	Go Live Mitigation
32	Physicians	In the case of an elective admission, the physician will need to remotely enter the patient's chart and record all of the patient's clinical documentation, and hence physicians will need to have permission for this type of access, and will also need to be trained on how to access and how to enter the correct document created for this admission.	Medium	Go Live Mitigation
33	Physicians	To prescribe laboratory test requests, a large number of clicks are required. It is necessary to format the tools that the system offers to mitigate this impact.	Medium	Go Live Mitigation
34	Physicians	On patient admission, physicians need to access a screen to perform patient medication reconciliation, and they will need to fill out what the patient already takes at home. However, filling in this reconciliation screen is a new activity, and the physician needs to be trained to use this functionality.	Medium	Go Live Mitigation
35	Physicians	The system does not offer a functionality to facilitate the "desensitization" prescription process and the oncologists will have to prescribe item per item, which in turn will generate an increase in workload.	Medium	Go Live Mitigation
36	Physicians	Intravenous and oral chemotherapy had different well-defined workflows and release for both. However, due to the electronic health record, the process will be entirely changed and physicians will have to be trained.	Medium	Go Live Mitigation
37	Physicians	New functionality in the system for the physician. The physician will need to register in the electronic health record which medications should be given to the patient to take at home upon discharge.	Medium	Go Live Mitigation
38	Physicians	Change process for the physician who will need to be trained on how to prescribe the ordering lab tests priority, that is, routine or urgent.	Medium	Go Live Mitigation
39	Physicians	New process for physician co-signing, which does not currently exist. Physicians will be trained to receive and sign the co-signatures sent by nurses and physiotherapists.	Medium	Go Live Mitigation
40	Physicians	There is a new anesthesia physician activity at the post-anesthesia recovery time, where there will be a pre-configured care and medication package, which requires activation. Physicians need to learn this functionality.	Medium	Go Live Mitigation
41	Physicians	Hospital discharge scheduling for the physician is a complex activity in the system that will demand more time with an increased workload.	Medium	Go Live Mitigation
42	Physicians	Activities usually done by the physician on a daily basis when performing non-surgical procedures will need to be recorded in structured fields in the system, with an increased workload.	Medium	Go Live Mitigation
43	Physicians	Increased workload for anesthesiologists due to the time required to record all the procedures performed, many in a structured field, in the anesthesia documentation.	Medium	Go Live Mitigation
44	Physicians	Considerable increase in phisician workload due to the complex process within the anatomopathological exam system request due to many structured fields to be filled in.	Medium	Go Live Mitigation
45	Physicians	New medication reconciliation discharge system activity with increased physician workload at the discharge process.	Medium	Go Live Mitigation

continue...

...Continuation

Table 1S. Description of impact according to professional group, criticality level and status

	Professional group	Description of impact	Criticality level	Status
46	Physicians	At the post-anesthesia recovery, if there is no integration of the infusion pump with the patient's electronic medical record, the continuous infusion medication information registered on the infusion pump will need to be manually entered by the physician into the system, increasing the physician'sworkload.	Medium	Go Live Mitigation
47	Physicians	At present, the physician usually prescribes a drug by trade name, whereas in the future they will have to prescribe drugs according to the system search option, and this may increase the uptime.<br>If the physician types in the drug name, the system search will only show results based on drugs by active ingredient, and if the physician starts typing by trade name, it will only bring up options by trade name.	Medium	Go Live Mitigation
48	Physicians	Increased workload for the physician auditors, as they will have to consult the patient's electronic health record, the legacy systems and paper information.	Medium	Go Live Mitigation
49	Physicians	The lab tests hierarchical visualization architecture in the system is not in accordance with the physician's reasoning and with the way the physician usually looks for exams, and this will cause difficulties for the medical team when looking for lab tests results.	Medium	Go Live Mitigation
50	Physicians	Requesting microbiology tests in the system requires filling out many fields, which is a time-consuming process for the physician with an increased workload	Medium	Go Live Mitigation
51	Physicians	Non-standard drugs prescription will require the physician to fill out a form in a system, which will burden the physician's time.	Medium	Go Live Mitigation
52	Physicians	Prescription drugs that do not have preconfigured order sentences in the system will have to be filled in field by field, and this functionality is not easy, burdening the physician's time.	Medium	Go Live Mitigation
53	Physicians	Physician radiologist process change in the act of prescribing medications for patients during procedures impacting on the physician radiologist's workload.	Medium	Go Live Mitigation
54	Physicians	Searching for patients only by first name is not possible, which will burden the physician in time when locating a particular patient, and hence it is necessary to train the physician to search by last name.	Medium	Go Live Mitigation
55	Physicians	In the system, the anesthesiologist's work process for filling out the anesthetic chart is completely different to how it is currently done, which, in turn, requires training and is time-consuming.	Medium	Go Live Mitigation
56	Physicians	When searching for patients who need anesthesia for surgery or for procedures, the anesthesiologist will find that both items of information are on the same list, requiring that they perform filters, and affecting the physician's time.	Medium	Go Live Mitigation
57	Physicians	Process change in monthly dialysis reporting requiring the physician to take some actions in the system to include the information on the necessary lab test results in the report. Process change is time-consuming.	Medium	Go Live Mitigation
58	Physicians	Visualization of the dialysis patient's monthly report is sectioned according to professional category, making it difficult to consult the information. It is not possible to display the monthly report in a single view contemplating both medical information and multidisciplinary team information, making the physician's work difficult and increasing navigation time in the medical record to obtain the information.	Medium	Go Live Mitigation
59	Physicians	In the ICU the work is very dynamic and the physician accesses the medical record of the same patient a number of times. However, every time the physician has to access a patient's medical record, they will have to log in and log off, which is time-consuming for the physician.	Medium	Go Live Mitigation
60	Physicians	Change in the physician's way of working, both when entering diagnostic information and when consulting these same diagnoses. Today, ICU physicians are used to organizing diagnoses according to the severity order of problems. In the system, it will not be organized in this way.	Medium	Go Live Mitigation
61	Physicians	There are many non-standardized chemotherapy protocols in the institution, and for these cases a workaround solution is needed, because the prescription of non-standard medications in the system burdens the physician's workload with many clicks to fill out additional information as part of the authorization process.	Medium	Go Live Mitigation
62	Physicians	Increased physician workload when recording children's vaccinations due to the amount of information and the number of different fields to be filled in, which is time-consuming for the physician.	Medium	Go Live Mitigation
63	Physicians	In the system, inhalation prescriptions are complex to be filled in, which is time-consuming for the physician.	Medium	Go Live Mitigation
64	Physicians	Neonatologists will need to adapt to prescribing drugs which need dilution, because it is necessary to prescribe item per item, which is time-consuming for the physician.	Medium	Go Live Mitigation
65	Physicians	The system does not have the necessary layout for home prescriptions as per Brazilian standards. The layout will need to be developed according to what is culturally done in Brazil.	Medium	Go Live Mitigation
66	Physicians	Visualization of anatomopathological results in the system is complex and will demand additional time from the physician when searching for results.	Medium	Go Live Mitigation
67	Physicians	The motor physical therapy prescription in the system requires the physician to fill out some mandatory fields, which will demand more time from physicians.	Medium	Go Live Mitigation
68	Physicians	Viewing a patient's history requires navigating through a number of different screens, which will demand more time time from physicians.	Medium	Go Live Mitigation

continue...

...Continuation

Table 1S. Description of impact according to professional group, criticality level and status

	Professional group	Description of impact	Criticality level	Status
69	Physicians	Change in the hospital discharge process for the physician who previously only had to fill out one document that functioned both as a discharge report and a prescription. In the new system, they will have to fill out separate documents, increasing the physician's workload.	Medium	Go Live Mitigation
70	Physicians	The neonatologist physician will depend on the information placed by the obstetric center nurse in order to follow through with the admission process in the Neonatal ICU, seeing as the information migrates from the obstetric center to the Neonatal ICU. Neonatologists need to be advised that if the patient information has not been filled in correctly at the obstetric center, this will impact their work.	Medium	Go Live Mitigation
71	Physicians	After the system implementation there will be a legacy with patients' entire history on another system. Most of the information will not migrate to the new system and the consultation of historical information will not be easy to view.	Medium	Go Live Mitigation
72	Physicians	New physician activity where they must fill out a form with the medications that the patient will receive from the hospital to take home, increasing their workload.	Medium	Go Live Mitigation
73	Physicians	If physicians cannot find a preconfigured order sentence that fulfills the necessity, they can choose an order sentence and modify it. However, this action is not necessarily straightforward, taking up the physician's time.	Medium	Go Live Mitigation
74	Physicians	For an electrocardiogram request in the emergency unit a change in the process is necessary, so as not to interfere with door-to-electrocardiogram time. New activity for the physician.	Medium	Go Live Mitigation
75	Physicians	The way a cardiotocography image is visualized will be different from the way it is done today, so gynecological physicians need to be warned and trained.	Medium	Go Live Mitigation
76	Physicians	Instead of the physician directly accessing the patient's chart as the home screen, they must access a home screen with pending activities, which is not the physician's usual routine at present.	Low	Go Live Mitigation
77	Physicians	When viewing the medication schedule, there will be symbology not previously used in the current paper process. Physicians need to be warned and trained.	Low	Go Live Mitigation
78	Physicians	Writing an admission prescription with all the patient's medication needs for his treatment during hospitalization involves many clicks, and it generally takes a long time to make a complete prescription. It is necessary to format the functionality offered by the system.	Low	Go Live Mitigation
79	Physicians	Electronic medical records do not allow for correction, cancellation or deletion of information in a finished document. In these cases, it will be necessary to create an extra document.	Low	Go Live Mitigation
80	Physicians	Increased workload for the physician who will have to inform, in the electronic health record, whether the patient takes medication at home, and if patient has brought it to the hospital to be used during hospitalization.	Low	Go Live Mitigation
81	Physicians	New physician activity in outpatient care regarding cases with a referral indication for the emergency room, the physician will now record this referral on a form in the patient's electronic health record, called "pre-arrival form".	Low	Go Live Mitigation
82	Physicians	The co-signing process for the physician is a new process and the professionals will have to be trained mainly on how to refuse a co-signing act, because refusing the co-signing fact does not mean that the prescription will be canceled, they are different actions in the system.	Low	Go Live Mitigation
83	Physicians	New process for the physician, when prescribing antibiotics for a period, *e.g.*, 7 days, as the system will automatically suspend treatment after this period. Attention is required in case the physician wants to extend the treatment time.	Low	Go Live Mitigation
84	Physicians	In urgent cases, there will be a new physician process that requires physiotherapy prescriptions to be co-signed. In this situation, physiotherapists can prescribe and send a co-signature to the physician.	Low	Go Live Mitigation
85	Physicians	There is a tool that is a fill-in form and will be used for filling in clinical data. This tool does not allow you to minimize the window displaying the health record and then maximize it to finish filling it out. When you open this form type, it has to be filled in, finalized, and saved.	Low	Go Live Mitigation
86	Physicians	The death registration in the patient's prescription is a new physician activity in the system, which culturally does not place the death as a "prescription order". But from the recording information point of view, placing it in a structured field is the best way to trigger the actions and tasks arising from this situation.	Low	Go Live Mitigation
87	Physicians	Process change and culture change for the physician prescribing enteral diet, as the enteral diet prescription will be displayed along with medications.	Low	Go Live Mitigation
88	Physicians	The immediate start prescription requires signaling "T/N", which means "today/now" for this to occur. This is a new process for the physicians.	Low	Go Live Mitigation
89	Physicians	The system has a feature that is a benefit to the physician, which are the pre-configured prescription packages for specific diagnoses. However, the physician needs to be trained because the act of signing the package does not mean that it automatically starts to be valid. To become valid, you need the action "start".	Low	Go Live Mitigation
90	Physicians	Completely new process for the clinical staff where the food supplements will be entered into the system by the nutritionist and the physician will have to co-sign.	Low	Go Live Mitigation
91	Physicians	Process change for physician auditors who will have to learn to consult different screens and navigate the system, not only for physicians' documentation, but also for nursing and multiprofissional documentation too.	Low	Go Live Mitigation

continue...

...Continuation

Table 1S. Description of impact according to professional group, criticality level and status

	Professional group	Description of impact	Criticality level	Status
92	Physicians	Cultural change in the pediatrician's way of working when prescribing drug dosages in the form of oral drops in the system. As there is no drop/kilogram form, the physician will have to prescribe the total of drops needed.	Low	Go Live Mitigation
93	Physicians	The transfer prescription requires some filling mandatory fields that will demand more phisician time.	Low	Go Live Mitigation
94	Physicians	The process of prescribing medication requires many clicks and then there is a box with a specific icon to click on and conclude the action, demanding more of the physician's time.	Low	Go Live Mitigation
95	Physicians	At present, hematologists register information on clinical care in the blood bank's legacy software. However, in the new system, hematology physician will have a new document for recording hemotherapies, as required by the system.	Low	Go Live Mitigation
96	Physicians	New activity for the physician who will have to learn to handle the new electrocardiogram device that integrates with the new system.	Low	Go Live Mitigation
97	Physicians	The way of visualizing the days from newborn to 72hs will be different than it is done at present. Therefore, neonatal physicians need to be warned and trained.	Low	Go Live Mitigation
98	Physicians	The vaccine is administered by the nurse according to the vaccination schedule and there is no need for a doctor's prescription. However, the system requires a medical prescription, which burdens the physician with an unnecessary workload. It is necessary to find adequacy in the system to mitigate this impact.	High	Solved
99	Physicians	For clinical documentation in the system, the physician will need to be advised and trained on how the system works so as not to make mistakes and not to record information incorrectly. The physician needs to put the information on screens that will then migrate to a document. The impact is even greater in the case of "surgical description", as situations may arise where a surgery that was programmed in the system may change because of clinical conditions. Some system solution or some process solution must be found to prevent this.	High	Solved
100	Physicians	Depending on where the physician places the diagnoses information, it can be archived and may not be available on the patient's chart. This process needs to be reviewed and corrected.	High	Solved
101	Physicians	The path for canceling diagnostics that may have been misplaced is difficult, requiring many clicks.	High	Solved
102	Physicians	There is no field available to put the patient's religion, and according to the institution's values this information is important to have in the patient's chart header.	High	Solved
103	Physicians	Today, medical oncologists use a drawing of the human topography for noting where the tumor or lesion is. As the system does not display any kind of drawing, oncologists will have to describe the location in writing.	High	Solved
104	Physicians	The system does not allow prescribing the loading dose and the maintenance dose for the same infusion bag. This impacts the physician's work. It is necessary to find some bypass solution in the system.	High	Solved
105	Physicians	There are situations where physicians prescribe a drug that should only be administered together with another specific drug. The problem is that the drugs are organized in alphabetical order and this hinders the reading of physicians, especially in case physicians decides to suspend it, as they may suspend a drug and not realize that they will also have to suspend the other one. These two pieces of information are not close to each other in the prescription distribution.	High	Solved
106	Physicians	Pediatricians are used to prescribing the drug dilution, but seeing that with the new system some drugs will have a standard dilution, this changes the activity of the pediatrician who will have to perform new calculations to prescribe the drugs.	High	Solved
107	Physicians	For drugs that do not have standard pharmacy dilution, the pediatrician will have to access the calculator, add a diluent, calculate the infusion speed, etc. This burdens the pediatrician, increasing both their time and workload. It is necessary to check options in the system to solve this problem.	High	Solved
108	Physicians	At present the legacy system informs the physician throughout patient care time what items are not covered by the health plan. The physician contacts the administrative team and the patient is notified. The new system does not have this action. This system functionality needs to be reviewed, as it compromises the physician's work and the institution's image.	High	Solved
109	Physicians	The CBC result visualization is culturally very different from the one available in the system. It is necessary to discuss the system presentation for this test result, as using this default screen will demand a very big cultural change for physicians.	High	Solved
110	Physicians	For drugs that require dilution in serum, the system has two options for presentation form, but only one can be chosen, either adding the final volume or adjusting the final volume. Therefore, it is necessary to choose between the presentation used by the pediatric area (adjusting the volume) or the presentation used in the adult patient area (adding the volume). Either choice will impact the other medical area.	High	Solved
111	Physicians	Today, when the physician prescribes a drug and there is no drug in stock, the administrative professional notifies the physician, changes it to a substitute drug that is in stock (same drug but from another manufacturer) for the physician and the physician only signs it off. With the the new system, physicians will be notified and they need to make the change themselves, thus increasing their workload.	High	Solved
112	Physicians	In the new system, bedside tests collection, such as blood glucose strips, requires the pathologist signature, who will have an additional activity of signing for these tests in the system.	High	Solved
113	Physicians	Prescribing saline 0.45% in the system will be a very difficult process, because it requires a serum that requires preparation (customization). A prescription will have to be made with half saline 0.9% and half distilled water, requiring many clicks and increasing the physician's workload. It is necessary to discuss a workaround solution.	High	Solved

continue...

...Continuation

Table 1S. Description of impact according to professional group, criticality level and status

	Professional group	Description of impact	Criticality level	Status
114	Physicians	The lab tests collection management in the institution is currently done by the nurses; the new system requires that the pathologist physician direct the collection, which requires a change in the process and overloads the physician with a task that is currently done by the nurses. It is necessary to find adequacy in the system for this process.	Medium	Solved
115	Physicians	Complex functionality in the system for future medical prescriptions, via remote access, for patients who would be admitted electively. Physicians need to be trained.	Medium	Solved
116	Physicians	The record of procedures and surgeries must be entered into the system according to the national coding table "TUSS". It is a new activity that will affect physicians' time, and it is necessary to check for alternatives.	Medium	Solved
117	Physicians	When the physician needs to refer a patient for an evaluation or consultation in another specialty, they usually fill out the clinical information documentation. In the system, they will have to write a prescription. There will be a process change and an increase in workload.	Medium	Solved
118	Physicians	At the checkup sector, when physicians make referrals, they write these on the checkup report. The process in the sector is changing at the moment and, with the new system, a specific document will have to be built to be filled out according to the sector's needs.	Medium	Solved
119	Physicians	The process of triage and patient care in the emergency room for physicians will require adjustments, because the system use for quick registration or full registration will depend on the emergency room demand. If it is full, fast registration mode will be used, whereby first the physician cares for a patient and then finishes the registration. If it is quiet, the system will perform full registration and then the physician cares for the patient	Medium	Solved
120	Physicians	The way to view image exams for the physician due to the new system will be complex, requiring a number of clicks and a specific icon to click on. It is necessary to review the integration and improve this process which should be automatic, where the physician simply clicks and opens the exam on the viewing screen.	Medium	Solved
121	Physicians	New physician pathologist activity to have the anatomopathological specimen releasing into the system. It is necessary to discuss whether this activity will remain with physicians or whether there is a workaround solution.	Medium	Solved
122	Physicians	Today there are packages for requesting lab tests that are ready-to-use. In the system, the physician will need to prescribe item per item. It is necessary to evaluate if any system functionality meets this demand so as not to impact on the physician's workload.	Medium	Solved
123	Physicians	When the patient goes to the operating room, in the system, it will be necessary to temporarily suspend the floor prescription, otherwise the medications will remain listed as overdue. It is necessary to find a solution so as not to burden the physician with the additional action of having to temporarily suspend the floor prescription.	Medium	Solved
124	Physicians	Nowadays, there is the bed reservation flow requested by the physician. In the new system there is a function called "scheduled transfer", but it does not work as it should. So, it is necessary to discuss and design this process in the system so that the flow is maintained and does not impact on the physician's work.	Medium	Solved
125	Physicians	To prescribe special control drugs for home administration in the new system, the physician will have to make filters and make many clicks, increasing the workload for the physician. It will be necessary to check if there are any settings that will make this path easier.	Medium	Solved
126	Physicians	The double checking of blood bags in the surgical center is done by the anesthesiologist together with the nurse. As there are many bags in the surgical center, this process is very time consuming and has a great impact on the anesthesiologist's work.	Medium	Solved
127	Physicians	Today, when requesting a laboratory test the physician does not need to specify what material type is being sent to process the test. For example, in the case of blood glucose, the physician does not need to inform whether it is plasma or serum, only the blood glucose is requested, which is a blood test. This creates an impact for medical staff with new activity and process change, thus a solution needs to be discussed.	Medium	Solved
128	Physicians	Medications administrated by the anesthesiologist in the operating room will not be visualized in the prescription in the system. This process needs to be discussed to find a solution because the information on what was administered in the operating room needs to be displayed in the prescription for the patient's physician, and not only in the anesthesia documentation.	Medium	Solved
129	Physicians	In cases where a continuous infusion with medication and a standard dilution runs out of stock, it is necessary to verify how it will be replaced, because if the exchange solely depends on a physician's prescription, it will burden the physician in workload and impact the patient care flow.	Medium	Solved
130	Physicians	The anatomopathological specimens that are discarded need to be analyzed even when being discarded. Even though nowadays physicians have the option of not requesting analysis for specimens that are discarded, the system requires the specimen analysis of any material, and the institution understands that this procedure is necessary because it is a matter of safety for the patient. It will be necessary to design this workflow.	Low	Solved
131	Physicians	Some medications are taken at a very specific time, for example antibiotics 1 hour before surgery. These prescriptions are difficult in the system which requires many clicks. Some workaround solution has to be discussed in order not to burden the physician's workload.	Low	Solved
132	Physicians	The newborn day count is displayed differently in the chart header and within the clinical documentation workflow. There is a need for standardization and verification of a solution to the problem.	Low	Solved

continue...

...Continuation

Table 1S. Description of impact according to professional group, criticality level and status

	Professional group	Description of impact	Criticality level	Status
133	Physicians	The anesthesia type performed at delivery does not migrate from the anesthesia documentation to the obstetrics documentation. So, the obstetrics physician will have to enter the information manually, increasing their workload.	Low	Solved
134	Physicians	The pre-anesthesia evaluation form has structured fields, but if certain surgeries are not standardized, this information will not appear on the form and the anesthesiologist will not be able to enter this information, which will have a direct impact on their work. It is necessary to discuss an outline solution.	Low	Solved
135	Nurses	Activities performed by the physician nowadays, such as recording part of the mother's data, the Apgar score, etc. will be performed by the nurses.	High	Go Live Mitigation
136	Nurses	Some activities performed by the nurse in the obstetric center related to maternal conditions will now be performed 3 times in the newborn's electronic health record: at birth, at 24 hours, and at discharge. Note: it is necessary to check if the information pertains to the right newborn and the right mother.	High	Go Live Mitigation
137	Nurses	At the emergency unit, performing quick registration before triage is a new process, with an activity change. There will be a need for training because it will be done by the nurse in triage.	High	Go Live Mitigation
138	Nurses	For safety measures, nursing will have to record all information from the blood bag in the patient's chart, since the blood bank has a separate system. There will be an increased nursing workload in the case of blood component bags.	High	Go Live Mitigation
139	Nurses	Setting up continuous infusion medication and linking the infusion pump to the patient's electronic health record is a complex activity that demands more time and increased workload for nurses.	High	Go Live Mitigation
140	Nurses	If the physician chooses an inappropriate dosing frequency for one or more medications, the nurse will have to reschedule all the medication timetables for that particular medication, and the rescheduling of the entire sequence of timetables is laborious and will greatly burden the nursing staff's working time.	High	Go Live Mitigation
141	Nurses	New activity for the imaging sector nurses, who must fill out a form with patients' information which used to be done by patients themselves. This is a new process that also impacts the nurse's time.	High	Go Live Mitigation
142	Nurses	The visualization of the medication doses to be administered to patients is not intuitive. The drug name stands out, but not its dosage. This requires training and attention from nurses.	High	Go Live Mitigation
143	Nurses	The screens require updating to see if there are any new tasks to be performed. The nursing staff needs to be trained to get used to refreshing the screens if a patient's chart is open for too long.	High	Go Live Mitigation
144	Nurses	Usually nursing care and any intercurrences with the patients are documented. However, intercurrences that did not happen do not need to be registered, and supposedly if it is not registered it is because it did not happen. Therefore, in the new system, the nursing staff will have to compulsorily register that it did not happen, which is time-consuming.	High	Go Live Mitigation
145	Nurses	Increased nursing staff workload because every time they administer an intravenous medication, they will have to inform in which location it was administered, inputting the information into a structured field.	High	Go Live Mitigation
146	Nurses	Prescriptions prepared by the physician need to be started by the nurse in order to become effective. New nurses' activity due to process change.	High	Go Live Mitigation
147	Nurses	In the case of care in the elderly residence unit, when the institutionalized patient leaves to be with family, it is necessary to deactivate the patient's prescription and reactivate it again when the patient returns. New nurses' activity due to process change.	High	Go Live Mitigation
148	Nurses	In the hemodynamics sector, the special material used will have to be posted by the sector nurse, who will also have to request the physician's co-signature. New activity for the nurse sector.	High	Go Live Mitigation
149	Nurses	Change of process for nurses when they need to reschedule a task or a medication, which involves too many clicks, burdening their workload.	High	Go Live Mitigation
150	Nurses	In the nursing staff's process of administering medication, the standard screen mode only shows the next doses to be administered, but when they need to view doses already administered, it is necessary to navigate for longer, so as to find the information, impacting on the nursing staff's time.	High	Go Live Mitigation
151	Nurses	Process change in the case of medications that need double-checking to be performed where the two nursing professionals will have to perform this task and register on the patient's electronic health record together at the bedside. New nurses' activity due to process change.	High	Go Live Mitigation
152	Nurses	Process change for ambulatory nurses, because the form that was filled in by the patient on paper will now have to be filled out electronically in the system by the nurse.	High	Go Live Mitigation
153	Nurses	Increased workload for dialysis nurses when calculating Kt/V, as they will need to enter information field-by-field in structured fields.	High	Go Live Mitigation
154	Nurses	Process change for nurses who will need to prescribe vaccines and then check the vaccine administration. New activity with increased workload.	High	Go Live Mitigation
155	Nurses	New nursing process in the elderly residence unit for delivering medication to the patient in situations where they leave the institution temporarily, due to the new form in the system for delivery and orientation of the medication that the patient has to take at home.	High	Go Live Mitigation

continue...

...Continuation

Table 1S. Description of impact according to professional group, criticality level and status

	Professional group	Description of impact	Criticality level	Status
156	Nurses	Change in the process of printing labels to be put on lab tests collection tubes, as the presentation generated by the system is different from the way it is currently done and the nursing staff will have to be careful not to make errors.	High	Go Live Mitigation
157	Nurses	Cardiotocography will not be integrated into the system at this time, so the exam must be digitized for storage. New activity with increased workload.	Medium	Go Live Mitigation
158	Nurses	Increased nursing staff workload, because in addition to the new quick registration activity, nurses will also have to register the number and time of the password provided to the patient at the totem.	Medium	Go Live Mitigation
159	Nurses	Increased nursing staff workload, as they need to record the problems by searching a structured list in the system to trigger the tasks related to the specific care for that problem.	Medium	Go Live Mitigation
160	Nurses	In emergency care, in cases where the diagnoses require institutional protocols such as sepsis and stroke, there will be a new activity in the patient's electronic health record where the nurse will need to draw up these protocols with the physician's co-signature, increasing the nurse's workload.	Medium	Go Live Mitigation
161	Nurses	In situations where there is a need to correct an order for a lab test performed by a physician, the nurse will perform the correction and will need to send the co-signature to the physician. New activity, which increases nurses' workload.	Medium	Go Live Mitigation
162	Nurses	The process for emergency patient care must be adjusted. The care will be performed first and the information will be retroactively recorded in the patient's electronic health record, especially the medication times.	Medium	Go Live Mitigation
163	Nurses	New nurses' activity involving recording prescriptions made by physicians by verbal order and then also requesting a co-signature from physicians.	Medium	Go Live Mitigation
164	Nurses	Double-checking the process of administering blood bag on the patient is complex in the system and will increase nurses' workload.	Medium	Go Live Mitigation
165	Nurses	To increase the safety of recording materials and medications used in the operating room, the electronic health record has specific fields to be filled in. This is a nursing process change with an increased workload.	Medium	Go Live Mitigation
166	Nurses	Retroactive registration of medications performed in emergency care, such as in cases of cardiorespiratory arrest, is a complex activity in the system that increases the nursing staff´s workload.	Medium	Go Live Mitigation
167	Nurses	When medications prescribed by the physician need dilution, the nurse will have to record the medication dilution in the system. It is a new activity with increased workload and process change for nurses.	Medium	Go Live Mitigation
168	Nurses	Recording a patient's home use medications by the nursing staff requires access to a number of screens and many fields and requiring many clicks, which will make this action in the patient's electronic health record quite difficult.	Medium	Go Live Mitigation
169	Nurses	The nursing staff will have to adapt the passing duty process using the system, which will have a new activity with records of "past duties" and "incoming duties".	Medium	Go Live Mitigation
170	Nurses	The surgery center nursing team needs to register in the patient's chart the materials used in the surgery center, as well as register the surgery center's clinical documentation. The team is used to having this done simultaneously by two different professionals to gain agility, but the system does not allow two professionals to register information in the same document simultaneously. Professionals will need to be informed and trained.	Medium	Go Live Mitigation
171	Nurses	The imaging exams registration will need to be done in two different systems, increasing nurses' workload.	Medium	Go Live Mitigation
172	Nurses	If the physician is delivering care at a distance, the nurse can prescribe and generate a co-signature for the physician. However, the step by step for this process will demand more time from the nurse, because they need to comunicate the physician, prescribe, wait for the physician to sign it off in the system, so that they can then execute the order.	Medium	Go Live Mitigation
173	Nurses	In areas where the infusion pump does not integrate with the system, information from the pump will have to be manually entered into the system, compromising the nursing staff's time.	Medium	Go Live Mitigation
174	Nurses	New nursing staff activity for the venous access procedure, either access or withdrawal, whereby there is a lot of information to be placed in structured fields such as catheter type, laterality, and other fields, burdening the nursing staff work.	Medium	Go Live Mitigation
175	Nurses	New nursing activity where nurses will have to generate a report for high-cost materials used in the operating room, which requires a co-signature from the physician.	Medium	Go Live Mitigation
176	Nurses	Total process change for the nursing staff in the step-by-step checking procedure of anatomopathological specimens in the operating room to be sent to the laboratory. It is a new activity with increased workload.	Medium	Go Live Mitigation
177	Nurses	Process change for nurse auditors who will have to audit the accounts in the backoffice legacy system, as they already do, and will now have to audit the patient's electronic health record as well, generating increased workload.	Medium	Go Live Mitigation
178	Nurses	Process change for delivering medications to the patient upon discharge. A new form completed by the physician in the system will have to be checked and verified by the nurse before the medication is delivered to the patient, and there will be a final task that requires checking that it has been delivered.	Medium	Go Live Mitigation
179	Nurses	The billing process requires nurses to adapt, because there will be specific procedures depending on the items being charged.	Medium	Go Live Mitigation
180	Nurses	Change in process for the nursing staff, when a medication is not administered on time, some steps need to be taken in system to justify the non-administration of the dose.	Medium	Go Live Mitigation

continue...

...Continuation

Table 1S. Description of impact according to professional group, criticality level and status

	Professional group	Description of impact	Criticality level	Status
181	Nurses	In cases of continuous infusion, the checking process with completion of some structured and mandatory fields increases workload for nursing staff.	Medium	Go Live Mitigation
182	Nurses	When the patient is in a temporary location, such as the surgical center or image sector, nurses will need to register this information in a field in the system, and this field must be integrated with the legacy billing system in another field called "receiving location".	Medium	Go Live Mitigation
183	Nurses	Change in the process of recording clinical information (procedure history, alergies, etc.) for nurses, where there will be a single form for recording all information in structured fields, increasing the workload.	Medium	Go Live Mitigation
184	Nurses	Change in the process of recording clinical information for patient transfer, where there will be a specific form for recording all information in structured fields, increasing the workload.	Medium	Go Live Mitigation
185	Nurses	Nursing process change relating to all registers of the patient's controls such as vital signs, fluid balance, and others, seeing as at present they are all recorded in documents, whereas in the system they will be recorded in single screen with all the structured registers.	Medium	Go Live Mitigation
186	Nurses	Process change for outpatient nurses, who must record outpatient monitoring on a form in the system.	Medium	Go Live Mitigation
187	Nurses	New process for nurses in case of temporary location situations, whereby the nurse will have to change the patient's location correctly and record itens being charged only after the temporary transfer has been carried out, so as to avoid mistakes.	Medium	Go Live Mitigation
188	Nurses	The system-generated lab tests identification label for sticking on the vials has a larger size than the current size, which fits perfectly. A workaround is needed.	Medium	Go Live Mitigation
189	Nurses	The administrative discharge performed for long-stay patients is very complex, involves all care areas and also the backoffice area. This will require mapping the entire process and creating a new flow design in the new system.	Medium	Go Live Mitigation
190	Nurses	Although the electronic medical record promotes greater information security, the hospital's nursing leadership has chosen to maintain the process of double-checking prescriptions, which will increase the workload for nursing, and thus the institution must update its institutional policy for the electronic health record.	Low	Go Live Mitigation
191	Nurses	The probable delivery date calculation is done automatically based on the date of the last menstrual period entered in the system, but if the user makes a mistake, the system will do the automatic calculation and will not allow any changes.	Low	Go Live Mitigation
192	Nurses	In the case of a temporary patient transfer, such as displacement for an exam in some sectors or a surgery, nursing will have to adapt to record this process in the system, where the nurse will have to log in and change the patient's location.	Low	Go Live Mitigation
193	Nurses	New activity for nurses, who will have to record the exact time the patient arrives in the observation room of the Emergency Unit in the patient´s electronic health record, affecting the nurses's time.	Low	Go Live Mitigation
194	Nurses	The data from the monitors sent to the system must be validated by nursing to avoid unrealistic data cases due to an error in the device reading. The validation in the system is a time-consuming nursing task.	Low	Go Live Mitigation
195	Nurses	The process of checking oral suplements by the nurses will be done in a diferent location/screen than enteral diets, therefore when checking oral suplements nurses need to change screens in the system.	Low	Go Live Mitigation
196	Nurses	Process change for the maternity ward nurse regarding the probable delivery date registration in case of abortions or premature birth, because the nurse needs to stop the countdown in the system, otherwise the system will continue counting.	Low	Go Live Mitigation
197	Nurses	The new barcode reading process for medication administration is safer, but it will be necessary to verify how to check the medication when the flask has 400mg and the patient needs to receive 500mg, for example. The system will automatically compute two flasks, and therefore it is necessary to train nurses on how the system works in this situation, so as not to make errors.	Low	Go Live Mitigation
198	Nurses	The recording of patients' medication history in the nurse's documentation is complex and with many clicks, increasing the nursing staff's workload.	Low	Go Live Mitigation
199	Nurses	The system converts the infusion speed from 30min to 0.5 hours, which impacts all of nursing, seeing as the latter (0.5hs) is not common practice nowadays. It is necessary to verify the adjustment in the tool for changing the format to 30min.	High	Solved
200	Nurses	The insulin calculator in the system does not round up, leaving a result with many decimal places after the comma, which is unviable. The calculator functionality needs to be reviewed.	High	Solved
201	Nurses	In the system, when the patient goes to the operating room, it will be necessary to temporarily suspend the floor prescription, otherwise the administration of medications will remain listed as overdue. If the physician forgets this action, the nurse will have to perform the action, generating a request for a co-signature fromr the physician. It is necessary to find a workaround solution so as not to burden the nurse with this additional action.	High	Solved
202	Nurses	Nurses today have a procedure for administering and recording the infusion speed of intravenous medications. In the system, this process is different, as nurses have to register this information in a prescription and generate a request for a co-signature for the physician, increasing the workload for nurses.	High	Solved
203	Nurses	The visualization of patient bed charts for the nurse is difficult, and a solution must be found.	Medium	Solved

continue...

...Continuation

Table 1S. Description of impact according to professional group, criticality level and status

	Professional group	Description of impact	Criticality level	Status
204	Nurses	At present, the glycemic curve test generates the labels for all the collection times for nurses. In the new system, the nurse has to generate each individual label, which increases the nurses' workload. It is necessary to verify if it is possible to adapt the system.	Medium	Solved
205	Nurses	In the current discharge process, the physician leaves a request for laboratory tests called "collection for discharge" and the nursing team collects at scheduled times, as part of the sector's routine. However, in the system, this request would demand a special schedule that is different from the routine schedule, which would impact the nursing staff's workload. It is necessary to review this process.	Medium	Solved
206	Multiprofessional Team	Process change for nutritionists who will have to register clinical information in the patient's electronic health record, and also access the legacy diet control system.	High	Go Live Mitigation
207	Multiprofessional Team	Change in oral diet prescription process for nutritionists, where they will need to be trained to make prescriptions in the patient's electronic health record and no longer in the legacy diet management system.	High	Go Live Mitigation
208	Multiprofessional Team	Process change for nutritionists in the patient admission flow to the bed. When the physician prescribes the diet, the information will be sent automatically in the system to the nutritionist, and no longer via nurse's call phone. Therefore, nutricionists need to check if the patient is already in the assigned bed before visiting the patient.	High	Go Live Mitigation
209	Multiprofessional Team	New activity for the nutritionist who will have to check the prescription made by the physician in the system, and validate item per item.	High	Go Live Mitigation
210	Multiprofessional Team	New nutritionists' activity to record re-evaluations and to monitor diet prescriptions. Process change for nutritionists who previously tracked everything on the legacy diet system map.	High	Go Live Mitigation
211	Multiprofessional Team	Process change for the nutritionist who will have to update the clinical records in the system, but the information is structured and must be filled in field by field.	High	Go Live Mitigation
212	Multiprofessional Team	New activity for the milk bank staff, who will need to check milk delivery in the patient's electronic health record.	High	Go Live Mitigation
213	Multiprofessional Team	New activity for nutritionists who will have tasks to check, and this task checking process is new for the nutrition sector.	High	Go Live Mitigation
214	Multiprofessional Team	New activity for the physiotherapist who will now send a co-signature request to the physician in cases of changes in the prescribed therapy.	Medium	Go Live Mitigation
215	Multiprofessional Team	New activity for the physiotherapist to send a co-signature request to the physician in cases of emergency therapy prescription.	Medium	Go Live Mitigation
216	Multiprofessional Team	Process change for nutritionists who will have to prescribe the actual products related to the oral supplements or enteral diet prescribed by the physician.	Medium	Go Live Mitigation
217	Multiprofessional Team	New activity for nutritionists, who must now register food allergies in system, which previously were only registered in the legacy nutrition management system.	Medium	Go Live Mitigation
218	Multiprofessional Team	There will be form in the system that must be filled out by the multidisciplinary team. This is a change process for nutritionists, who will have to learn how to fill out their part in the form.	Medium	Go Live Mitigation
219	Multiprofessional Team	New physiotherapist activity related to checking the completion or not of each physiotherapy session. If the patient does not attend, it will be necessary to record the justification in a structured field.	Medium	Go Live Mitigation
220	Multiprofessional Team	Impact on the speech therapists work because when the physician prescribes evaluation or speech therapy treatment, this task does not go to any specific speech therapist. Therefore, these professionals will have to manage this flow and will have to assign themselves to the patient in order to provide the necessary care. New activity.	Medium	Go Live Mitigation
221	Multiprofessional Team	Physiotherapists will have to assign themselves to the patient in order to provide the necessary care. New activity of assigning and unassigning patients for physiotherapists.	Medium	Go Live Mitigation
222	Multiprofessional Team	Process change for nutricionists regarding the way patient lists are controlled on each floor, therefore they need to be trained.	Low	Go Live Mitigation
223	Multiprofessional Team	The outpatient nutrition agenda is a new functionality offered by the system that nutritionists will need to learn how to use.	Low	Go Live Mitigation
224	Multiprofessional Team	New nutritionist's activity concerning checking some scheduled procedures for billing.	Low	Go Live Mitigation
225	Biomedicals	The step-by-step protocol to process lab tests will not be available in the system. Therefore, laboratory biomedical staff will need to consult this procedure as needed, increasing their workload.	High	Go Live Mitigation
226	Biomedicals	The process of storing anatomopathological slides in the system is complex and may mislead the laboratory biomedical staff.	High	Go Live Mitigation
227	Biomedicals	New activity for imaging biomedical staff, due to the process change, when there is the need for correction of erroneously requested image exams, and the correction will have to be done by the biomedical staff.	High	Go Live Mitigation
228	Biomedicals	New activity for the imaging biomedical prescriber of contrast exams. They must also request the physician's co-signature via system, resulting in process change and taking up more time.	High	Go Live Mitigation

continue...

...Continuation

Table 1S. Description of impact according to professional group, criticality level and status

	Professional group	Description of impact	Criticality level	Status
229	Biomedicals	In the case of imaging exams where there is a drug prescription protocol, the biomedical doctor will have two new activities, prescribing the medication and sending a co-signature request to the physician.	High	Go Live Mitigation
230	Biomedicals	Tests labs processed in the flow cytometry department will require the laboratory biomedical's attention due to the process change with increased workload, in light of the information access that will require the biomedical specialist to access the patient's electronic health record if they need to compare current results with previous results.	High	Go Live Mitigation
231	Biomedicals	Change in the laboratory biomedical process related to test reports being released when there are tests that complement each other. This will demand new biomedical activity with increased workload.	High	Go Live Mitigation
232	Biomedicals	New laboratory biomedical activity, as they will have to select, when releasing the lab test report, whether the lab test was processed manually or automatically, affecting the biomedical's time.	Medium	Go Live Mitigation
233	Biomedicals	Change in the process of receiving test samples by laboratory biomedical staff, which will have different locations in the system for registration depending on the lab test. It will be a new biomedical activity and the workload will increase.	Medium	Go Live Mitigation
234	Biomedicals	Complex laboratory biomedical activity related to locating anatomopathological slides in the system.	Medium	Go Live Mitigation
235	Biomedicals	There is a laboratory biomedical attention point in the system, seeing that due to the access permissions, the laboratory sector can release tests from another laboratory sector in a non-interceptional way. It is necessary to choose filters with great attention.	Medium	Go Live Mitigation
236	Biomedicals	Increased laboratory biomedical workload, as they must now analyze and release results that are incompatible with life, because the system does not have an automatic lock for these situations, and requests a justification before allowing the release.	Low	Go Live Mitigation
237	Biomedicals	Tests are usually performed on equipment for consistently attaining correct laboratory test results. To ensure greater safety and quality, the test results of this equipment will integrate with the patient's electronic health record and this information will be available for consultation. However, this is a new process with new activities and increased laboratory biomedical workload.	Low	Go Live Mitigation
238	Biomedicals	As the system is American, some translations from English to Portuguese of system fields in the laboratory sector were not intuitive, which could compromise the biomedical staff's work. These field's translations need to be revised.	High	Solved
239	Biomedicals	The report on pending lab tests collected in all units and not yet received for processing in the laboratory is difficult for biomedicals to visualize in the new system, seeing as it is different to what is currently done. It is necessary to verify parameterizations in the system and check if it is possible to configure a report similar to the one that is done today.	Medium	Solved
240	Biomedicals	The report on pending lab tests results that have not yet been released is difficult to visualize for biomedicals, who will need to perform filters to visualize what they need, burdening the workload.	Medium	Solved
241	Pharmacists	It was detected that the system did not allow the pharmacist to see the stock medication at the moment of authorizing a dispensation of medication prescribed by the physician. This process needs to be reviewed, so as not to impact on the pharmacist's workflow.	High	Go Live Mitigation
242	Pharmacists	Medication delivery for the patient at the discharge time also changes the pharmacist's work process, seeing as there is a form in the system that is filled out by the physician, but which automatically triggers tasks for the pharmacist. These are new activities for the pharmacist.	High	Go Live Mitigation
243	Pharmacists	In cases when the nurse reschedules medication doses, the verification of this medication goes back to the pharmacists, increasing their workload.	Medium	Go Live Mitigation
244	Pharmacists	Drug stock records compiled by pharmacists requires completing many screens, affecting the pharmacist's working time.	Medium	Go Live Mitigation
245	Pharmacists	The process of checking drugs in stock and releasing them for dispensing is complex in the system and different from the way it is done at present, especially in emergency and surgical center situations. The change in the process will affect pharmacists' working time and they will need to be trained.	Medium	Go Live Mitigation
246	Pharmacists	With the system entry, there will be a drug administration barcode check, but when the product is manipulated and unitized by the pharmacy, a new label needs to be generated, increasing the pharmacist's workload.	Medium	Go Live Mitigation
247	Pharmacists	When dispensing drugs, there are new pharmacist activities within the system, where they need to "submit and apply" the drug and then proceed with the acceptance process, increasing their workload.	Low	Go Live Mitigation
248	Pharmacists	The drug stock visualization screen displays information on released and unreleased drugs all on the same screen, requiring the pharmacist to perform filters so as not to get confused, increasing their workload and possibly leading the pharmacist to error.	Low	Go Live Mitigation
249	Administrative team	New adminstrative activity for the maternity and obstetric center, which will now record all newborn data in the system in structured fields.	High	Go Live Mitigation
250	Administrative team	The lab tests report printing funtionality will show only one physician name on the report within a period of 24 hours, and if more than one physician has ordered lab tests, the system will not show the names of all the physians, it will only show the first name This impacts the administrative staff's routine. They will need to know about this change, especially the customer assistance staff, in case of patient complaints.	High	Go Live Mitigation

continue...

...Continuation

Table 1S. Description of impact according to professional group, criticality level and status

	Professional group	Description of impact	Criticality level	Status
251	Administrative team	Impact for the administrative staff in searching for patients in the electronic health record system, because the search process in the backoffice legacy is different from the electronic health record system, so they will need to be trained.	High	Go Live Mitigation
252	Administrative team	The billing process for items posted to the account late will change completely with the new system. There will be new activities for the administrative staff as a result of the process change.	High	Go Live Mitigation
253	Administrative team	Process change for the administrative staff in closing accounts for long hospitalizations, wherein the accounts are invoiced at the end of a period as predetermined by the institution, and if some items were not invoiced, the system issues an account report for late entries that will be under the audit sector's responsability.	Medium	Go Live Mitigation
254	Administrative team	In the case of outpatients, patient care administrative staff must assign a service number in the system before the patient even arrives at the outpatient clinic. Furthermore, in the case of private appointments, it is necessary to train the administrative staff to perform this procedure.	Medium	Go Live Mitigation
255	Administrative team	Increased workload for accounts receivable staff who will need to navigate through many screens in the system to obtain the necessary information.	Medium	Go Live Mitigation
256	Administrative team	Commercial sector administatives will have an increased workload as they will need to navigate through many screens in the system to obtain the necessary authorization request information from the paying sources.	Medium	Go Live Mitigation
257	Administrative team	Administrative staff process change in transplant surgery billing cases, where they will need to be trained.	Medium	Go Live Mitigation
258	Administrative team	Impact for the health records service coding team because these professionals already code data according to ICD table in two different systems, and they will also have to enter and navigate the electronic health record system and carry out the same process.	Medium	Go Live Mitigation
259	Administrative team	Printing lab test reports on the system requires navigating through many screens, as well as recording each lab test access number. The process is difficult, time-consuming, and will complicate the administrator's routine.	Low	Go Live Mitigation
260	Administrative team	Change in the process for administrative staff in cases of outpatient care scheduled image exams, because the system works with an estimated date and time of patient arrival, which is different from the current process.	Low	Go Live Mitigation
261	Administrative team	In cases of outpatient care, administrative staff will need to check in patients when they arrive for their appointment. There will be a process change with additional activity.	High	Solved
262	Administrative team	Viewing the lab test history report for laboratory administative staff will be difficult, and a workaround is needed.	Medium	Solved
263	Administrative team	For endoscopic exams where a biopsy was needed during the exam, at present physicians do not need to prescribe the biopsy, they just report it in the procedure description and the administrative professional launches the charge. In the new system it is necessary to evaluate and to define a new process for the administrative staff.	Medium	Solved
264	Administrative team	Deliveries at the obstetric center can happen with or without prior scheduling. As the new system requires pre-scheduling, the process needs to be redesigned so as to enable this functionality in the system.	Medium	Solved

Table 2S. Confirmation of predicted negative impacts

Expected impact	Impacts number n (%)
Confirmed	190 (89.20)
Not confirmed	23 (10.79)
Total	213 (100)
